# Supplementary material for: Spatiotemporal Patterns of Adaptation-Induced Slow Oscillations in a Whole-Brain Model of Slow-Wave Sleep
Source: Front Comput Neurosci. 2022 Jan 12;15:800101. doi: 10.3389/fncom.2021.800101 (PMC8790481; doi:10.3389/fncom.2021.800101)
Supplement: Supplementary file 1 [file Data_Sheet_1.PDF]

frontiers

Supplementary Material

1 SUPPLEMENTARY FIGURES

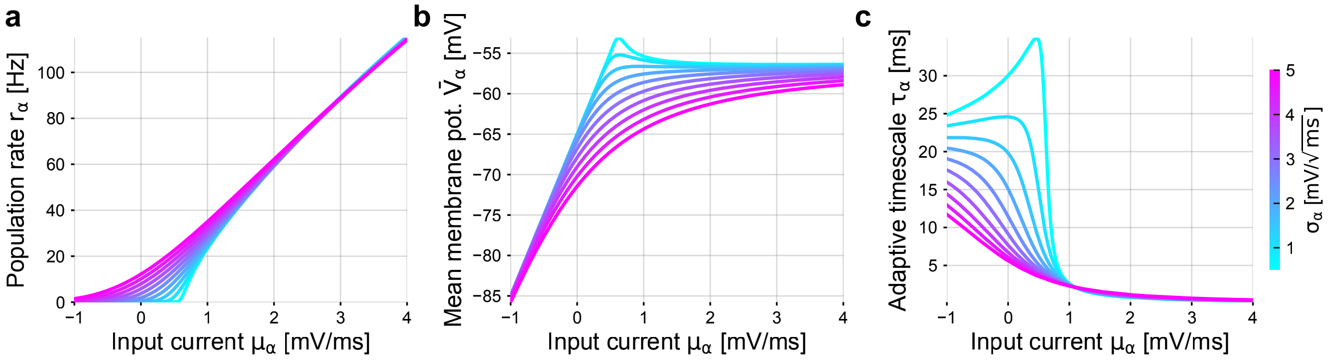

Figure S1. Precomputed quantities of the linear-nonlinear cascade model. (a) Transfer function for the mean population rate. **(b)** Transfer function for the mean membrane voltage. **(c)** Time constant τ_α of the linear filter that approximates the linear rate response function of AdEx neurons. The color scale represents the level of the input current variance σ_α across the population. All neuronal parameters are given in Table 1.

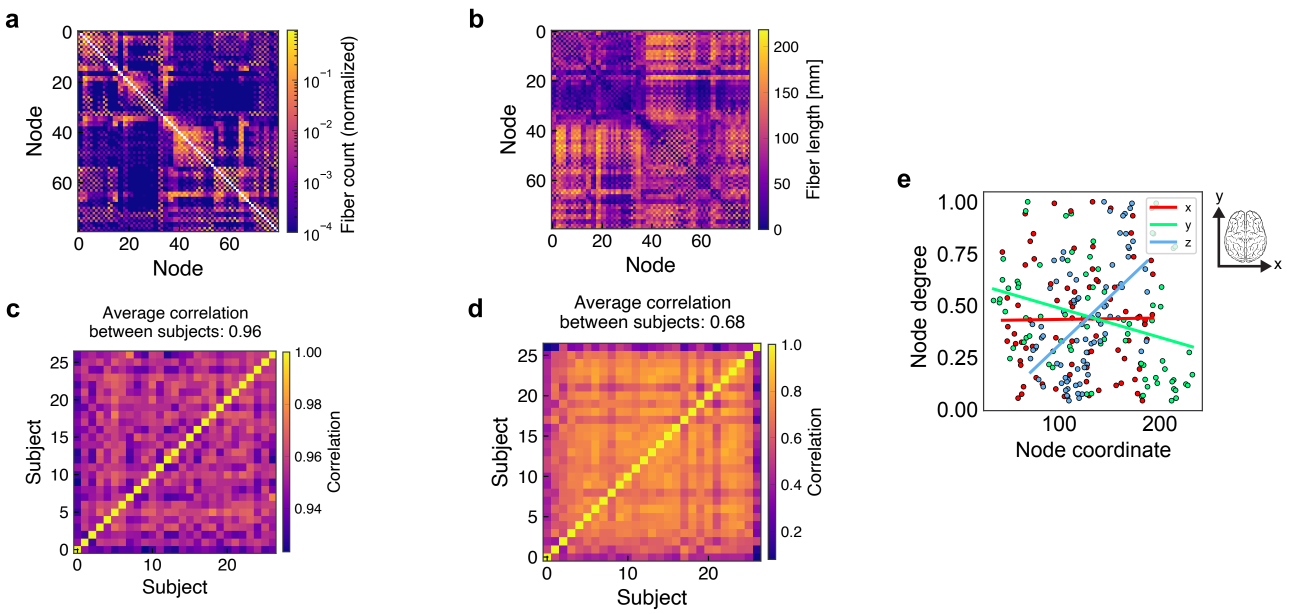

Figure S2. Structural brain data. **(a)** Structural connectivity matrix that defines the connection strengths between cortical regions from the AAL2 atlas. Colors indicate the number of fibers connecting any two regions, normalized by dividing by the maximum fiber count. The matrix is averaged across 27 subjects. **(b)** Fiber length matrix that defines the delay between nodes with the color showing the average length of the fiber bundle between any two regions. The matrix is averaged across all subjects. **(c)** Inter-subject correlation of the lower triangular entries of the structural connectivity matrices. The average inter-subject Pearson correlation of the individual structural connectivity matrices is 0.96. **(d)** Inter-subject correlation of the fiber length matrices. The average correlation is 0.68. **(e)** Heterogeneity of node degrees. The weighted node degree of every brain area is plotted against its three spatial coordinates. The x-direction (red) refers to the left-to-right axis of the brain, the y-direction (green) to the posterior-to-anterior, and the z-direction (blue) to the ventral-to-dorsal axis. Coordinates correspond to the center of mass of a brain region. Linear regression lines are shown for each direction. In the x-direction, the linear regression has an insignificant p-value such that no dependency can be observed. In the y-direction, the slope is -1.4×10^{-3} with $R^2 = 0.10$ and $p < 0.005$. In the z-direction, the slope is 4.6×10^{-3} with $R^2 = 0.28$ and $p < 0.001$.

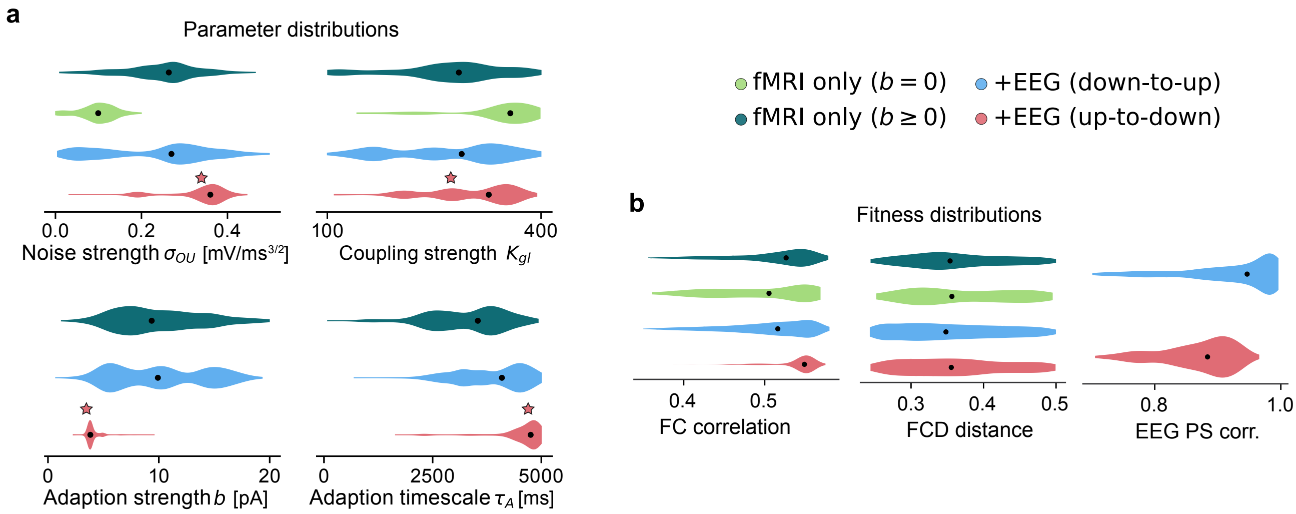

Figure S3. Optimization results. (a) Distributions of all all other optimized parameters across the final population of each optimization run additional to the input current strengths shown in Fig. 3 b in the main manuscript. Fit to fMRI data only without adaptation, $b = 0\text{pA}$, in dark green and with adaptation, $b \geq 0\text{pA}$, in light green. Fit to fMRI and EEG data with *down-to-up* solutions (blue) and *up-to-down* solutions (red). *Up-to-down* fits (red) have stronger noise σ_{ou} and weaker adaptation b compared to *down-to-up* fits (blue). Black dots show median values of the distributions. The star symbol indicates the parameters of the sleep model in Fig. 4 in the main manuscript. **(b)** Fitness distributions of the final population of each optimization.

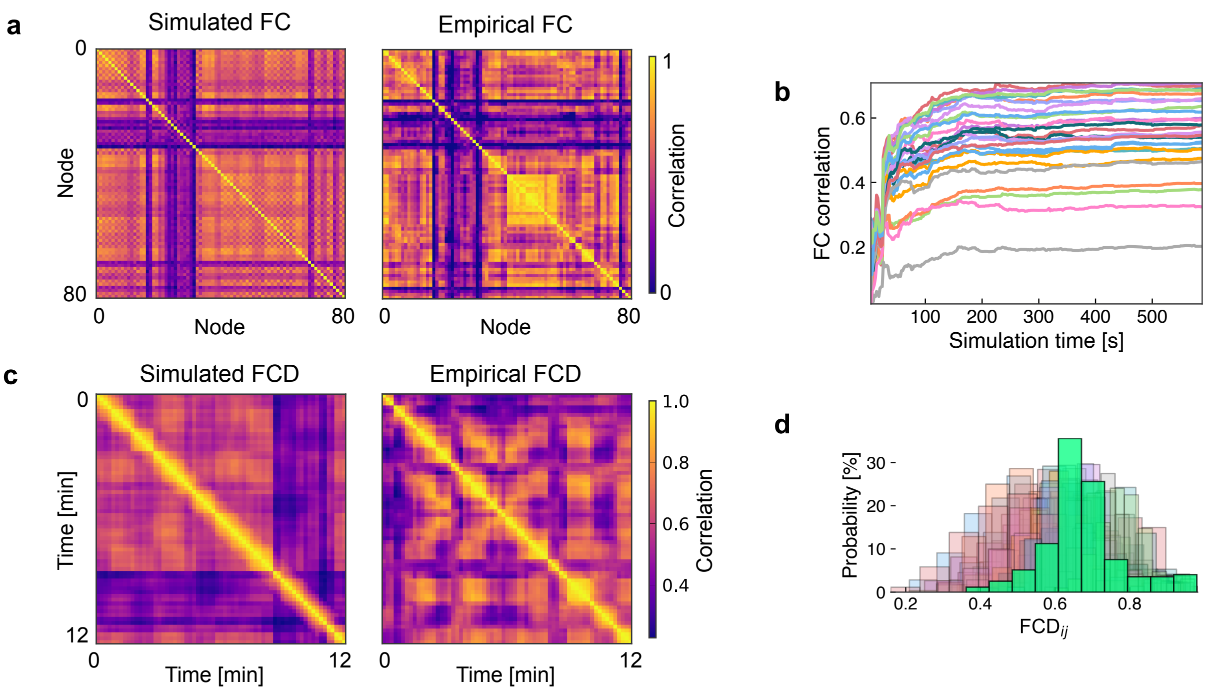

Figure S4. Model fit to fMRI data. (a) Simulated functional connectivity (FC) matrix of the sleep model and empirical FC matrix of the best fitting subject (from a total of 27 subjects). Color denotes the Pearson correlation coefficient of the BOLD time series for each pair of nodes in the brain network. Correlation between simulated and empirical matrices was 0.55 averaged across all subjects with the best subject reaching 0.70. **(b)** Correlation between simulated FC and all subjects FCs (different colors) as a function of the total simulation time. **(c)** Simulated and empirical functional connectivity dynamics (FCD) matrices. The empirical FCD matrix is shown for a the best-fitting subject. The Kolmogorov-Smirnoff distance between the distribution of the FCD matrix entries to the empirical FCD matrix entries averaged across all subjects was 0.28 with the best subject reaching 0.07. **(d)** Distributions of lower diagonal entries of the simulated FCD matrix (solid green) and the empirical data for each subject (different colors). Parameters are taken from the sleep model in Fig. 4 in the main manuscript (star in Fig. 3 b in the main manuscript and in Suppl. Fig. S3 a).

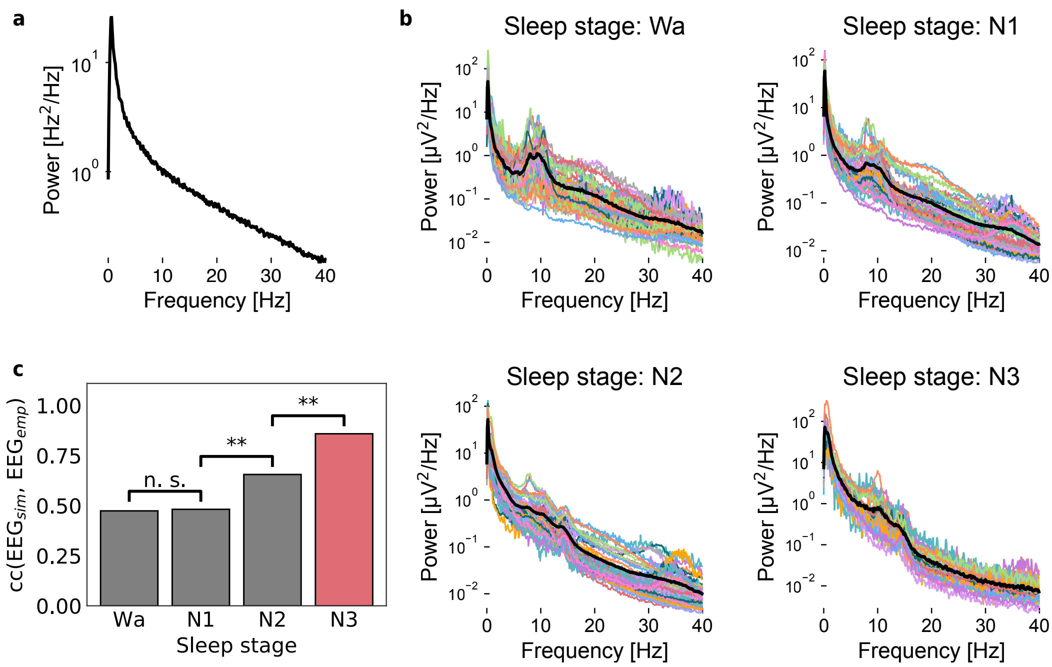

Figure S5. Model fit to EEG data. (a) Power spectrum of the mean firing rate of the sleep model (star in Fig. 3 b in the main manuscript and in Suppl. Fig. S3 a). **(b)** Empirical EEG power spectra of the awake state (Wa) and different sleep stages (N1-N3). Different colors denote the power spectra of the 18 different subjects. The black line denotes the across-subject average. **(c)** The model fits best to the empirical EEG power spectra during sleep stage N3 which was used as the target for fitting the model. The bars show the subject-averaged Pearson correlation coefficient (cc) between the power spectrum of the sleep model (EEG$_{sim}$) and the empirical power spectra of different sleep stages (EEG$_{emp}$). Sleep stage N3 is indicated in red. Means and standard deviations of the correlation coefficients are: 0.47 ± 0.11 (Wa), 0.48 ± 0.08 (N1), 0.66 ± 0.12 (N2), 0.86 ± 0.07 (N3). Parameters are taken from the sleep model in Fig. 4 in the main manuscript.

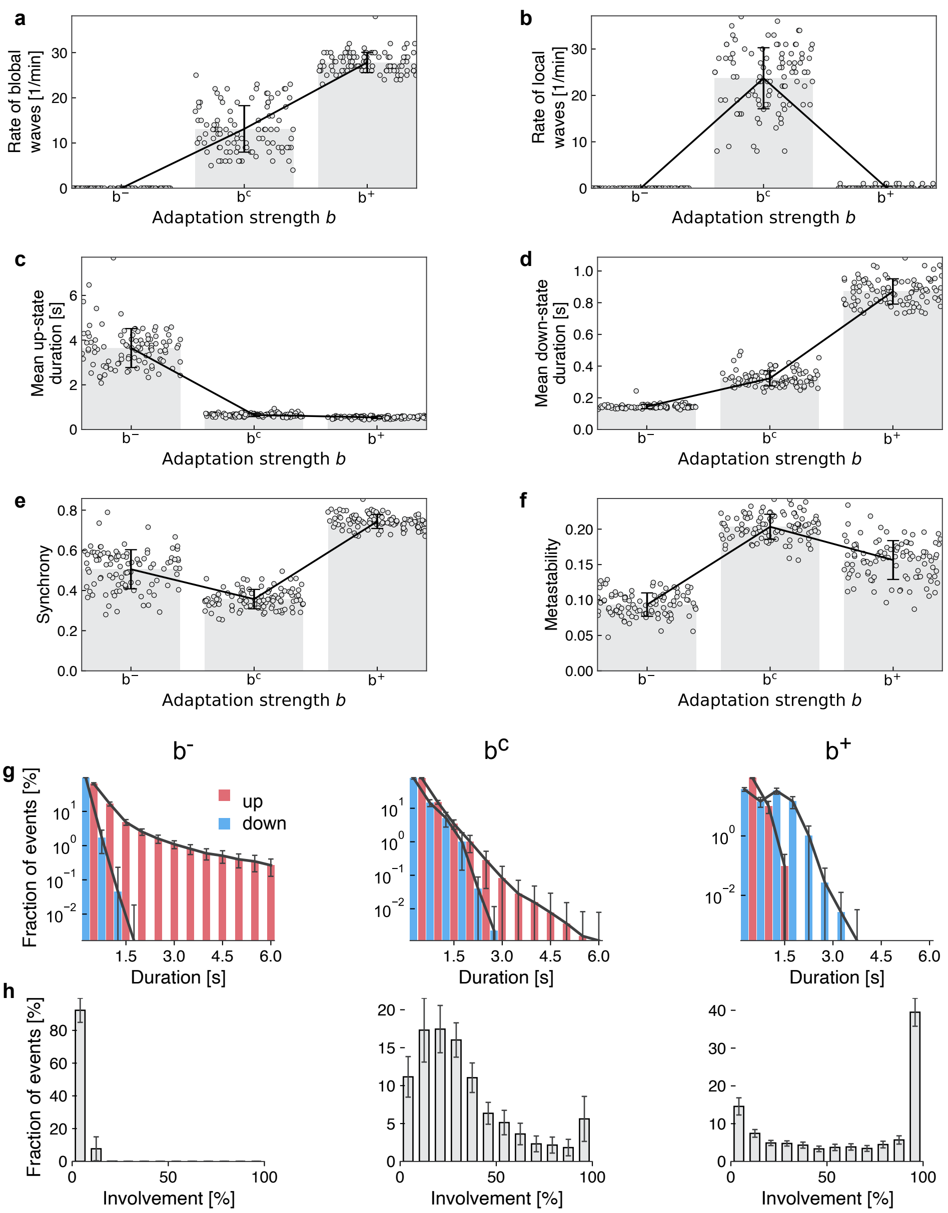

Figure S6. Population analysis of adaptation-dependent statistics. Analysis of the “up-to-down” population of sleep models shown in Fig. 3 b (red color), which were simultaneously fitted to fMRI and EEG data. The 100 best fitting individuals were analyzed (see Methods) for three different adaptation strengths: the their original (optimized value of the) adaptation strength parameter (b^c), a 50% reduction (b^-), and a 50% increase (b^+), resulting in a total of 300 individuals. The panels show: **(a, b)** The number of global (¿ 50**(c,d)** the mean duration of up-states and down-states, **(e,f)** the mean of synchrony and metastability, as measured by the Kuramoto order parameter, **(g)** the distribution of *up-state* (red) and *down-state* (blue) mean durations across time and brain regions (error bars indicate standard deviations) for three levels of adaptation strength b, and **(h)** the distribution of mean *down-state* involvement (error bars indicate standard deviations). All other parameters are as in Fig. 4 in the main manuscript.

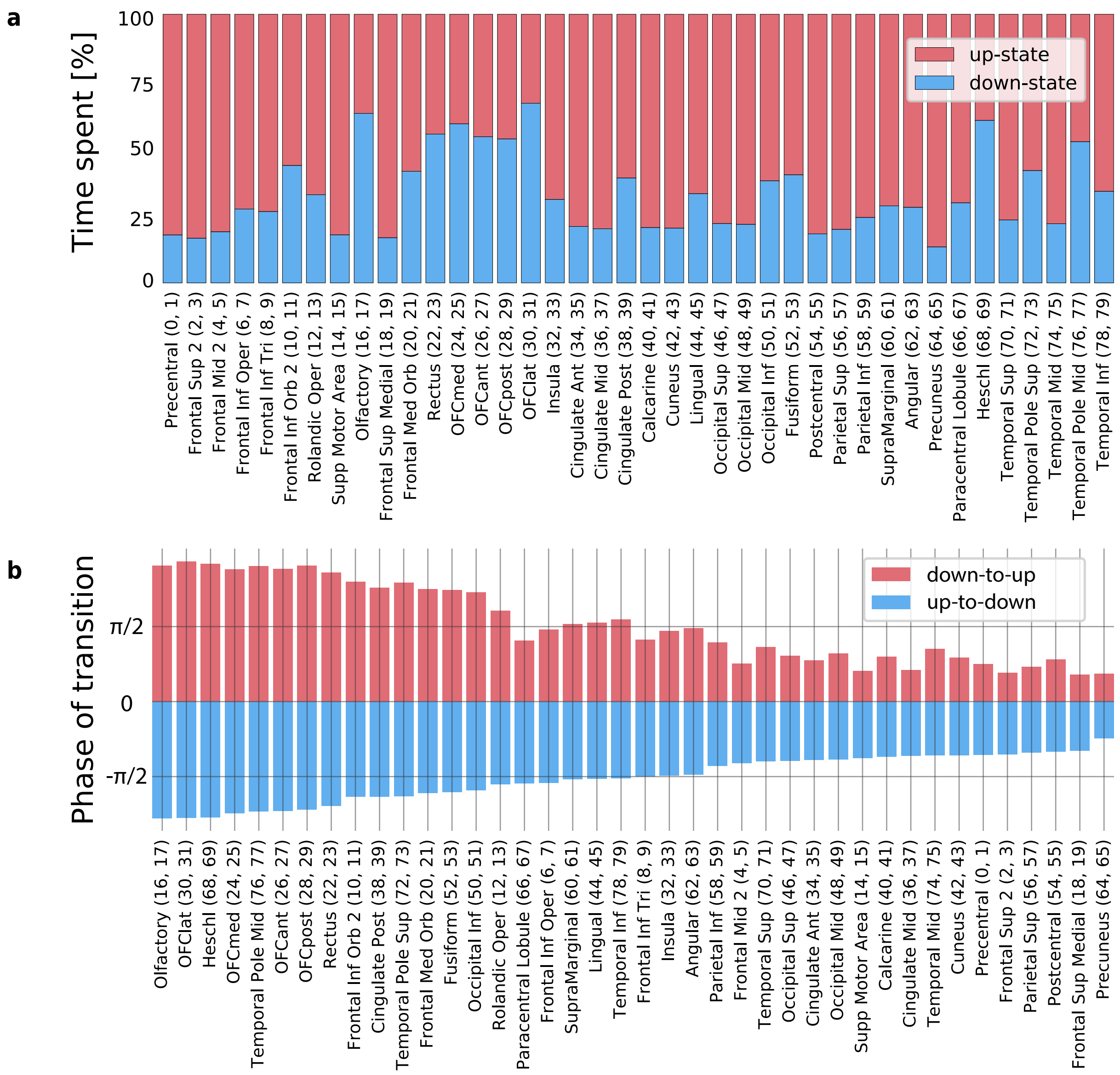

Figure S7. State durations and transition phases for the sleep model shown in Fig. 4 in the main manuscript. (a) Mean fraction of the duration of each brain area Rolls et al. (2015) spent in the *up-* (red) and the *down-state* (blue). The names and their AAL2 indices of all regions are shown on the x-axis. Durations are averaged across the corresponding contralateral regions. **(b)** Average transition phase of *down-to-up* transitions (red) and *up-to-down* transitions (blue) for each brain area, sorted by the mean transition phase to the *down-state*. Phases are additionally averaged across contralateral regions.

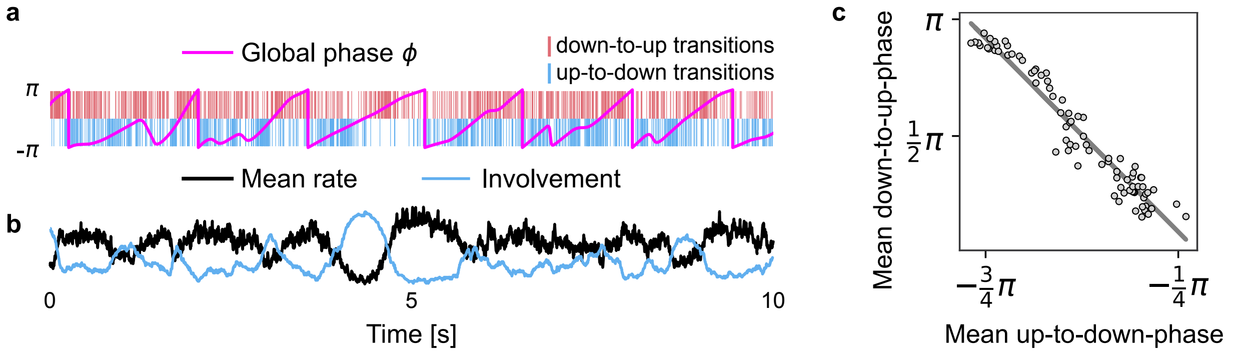

Figure S8. Involvement time series defines the global oscillation phase of an event with respect to a cortical *down-state*. (a) Global phase ϕ (pink) defined by the phase of the involvement time series superimposed with *down-state* (blue vertical lines) and *up-state* transitions (red vertical lines) of all brain areas. **(b)** Time series of *down-state* involvement (blue), measuring the fraction of brain areas in the *down-state*, and of the mean firing rate across all areas (black). **(c)** Brain areas that initiate *down-states* first, transition to *up-states* last. The mean *up-to-down* and *down-to-up* transition phases for each node in the brain-network are plotted against each other. The linear regression line has a slope of -1.76 ($R^2 = 0.92$ and $p < 0.001$). Results were obtained with the sleep model in Fig. 4 in the main manuscript.

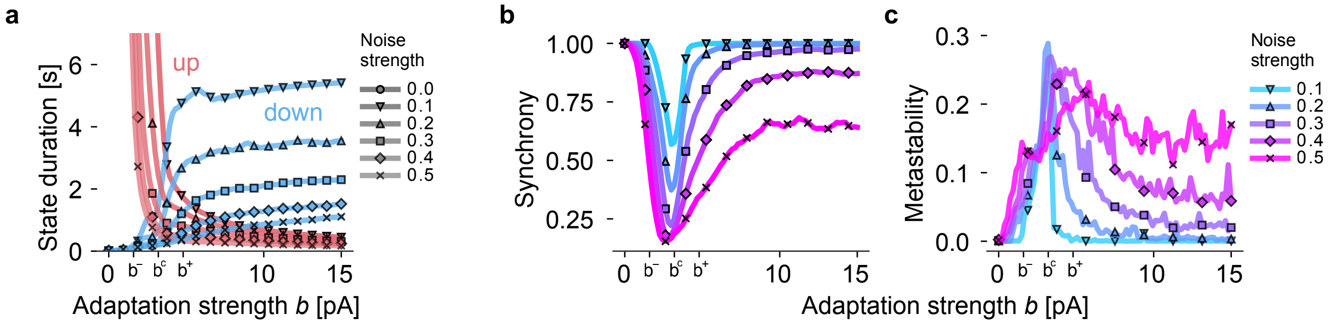

Figure S9. The statistics of slow oscillations depends on adaptation strength. (a) Average state durations for *up-* (red) and *down-states* (blue) as a function of the adaptation strength b and the noise level σ_{ou} (different lines). Noise strength is measured in $\mathrm{mV/ms}^{3/2}$. Tick marks b^-, b^c, and b^+ indicate the values for each panel in Fig. 5 a in the main manuscript. b^c denotes the best-fitting value of b obtained during the optimization procedure used in Fig. 4 in the main manuscript. **(b)** Synchrony of transitions to the *down-state* as measured by the temporal mean of the Kuramoto order parameter $R(t)$ (see Eq. 12 in the main manuscript). **(c)** Metastability defined as the temporal fluctuation of $R(t)$. Other parameters are taken from the sleep model in Fig. 4 in the main manuscript.

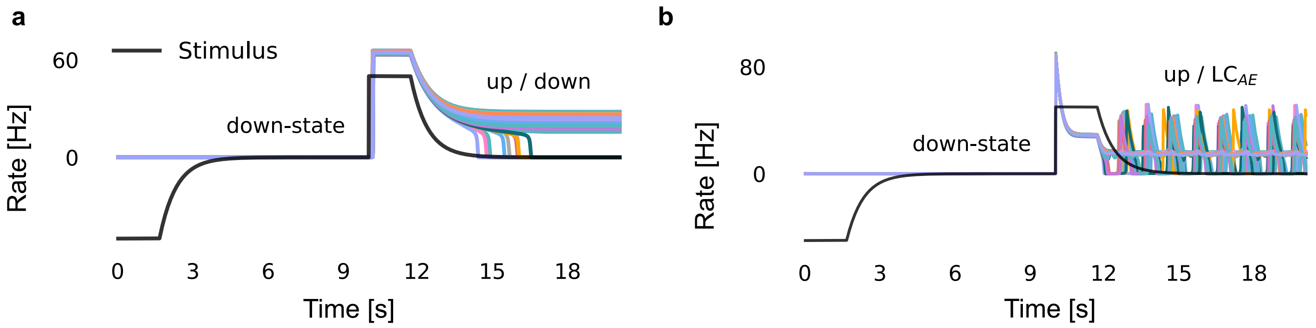

Figure S10. Network bistability. (a) The firing rate of each region is shown in different colors. Stimulus (black) pushes the system to the *down-state* and then to the *up-state*. Subsequent relaxation of the stimulus reveals partially bistable states, in which some regions remain in the *up-state* and other regions decay to the *down-state*. Parameters are $\mu_E^{\text{ext}} = 2.0\text{mV/ms}$, $\mu_I^{\text{ext}} = 3.5\text{mV/ms}\ \mu_E$ and $b = 0\text{pA}$. **(b)** Bistability between the *up-state* and the slow adaptation limit cycle LC_{AE}. Parameters are $\mu_E^{\text{ext}} = 2.8\text{mV/ms}$, $\mu_I^{\text{ext}} = 3.5\text{mV/ms}\ \mu_E$ and $b = 20\text{pA}$. Other parameters are the same as in the state space diagrams in Fig. 2 a in the main manuscript.

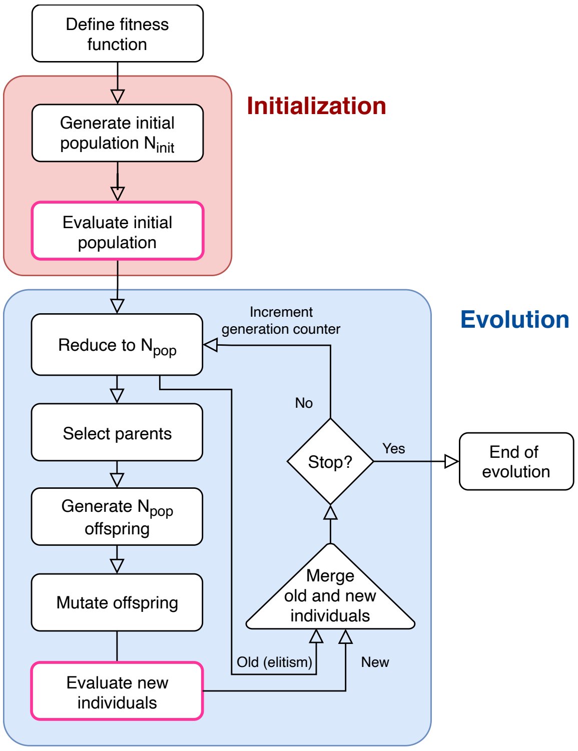

Figure S11. Schematic of the evolutionary algorithm The evolution is has two phases with its initialization phase shown in red and its second phase with repeating evolutionary rounds shown in blue. The evolutionary optimization stops when the generation counter reaches a predefined number of generations. The whole-brain model is simulated, and a fitness score is assigned in the pink boxes. N_{init} denotes the size of the initial population, and N_{pop} the size of the ongoing population. Details on the algorithm and the evolutionary operators used are provided in the Methods.

REFERENCES

Rolls, E. T., Joliot, M., and Tzourio-Mazoyer, N. (2015). Implementation of a new parcellation of the orbitofrontal cortex in the automated anatomical labeling atlas. *NeuroImage* 122, 1–5. doi:10.1016/j.neuroimage.2015.07.075
